# Effect of the Number of Gallate Groups of Polyphenols on the Structure, Gel Properties, and Biological Activity of Soy Protein Fibrils

**DOI:** 10.3390/foods14060974

**Published:** 2025-03-12

**Authors:** Tianhe Xu, Ruihan Su, Bowen Yang, Shicheng Dai, Junzheng Wang, Weixiang Zhu, Qi Fang, Huan Wang, Lianzhou Jiang

**Affiliations:** College of Food Science, Northeast Agricultural University, Harbin 150030, China; 15830825739@163.com (T.X.); neau_suruihan@163.com (R.S.); 13946087819@163.com (B.Y.); 18443602143@163.com (S.D.); 18245323025@163.com (J.W.); fqname@163.com (Q.F.)

**Keywords:** soy protein isolate, tannic acid, gallic acid, interaction mechanism, structure, gel property

## Abstract

Amyloid fibril hydrogels prepared via protein acid heating currently exhibit inadequate gel properties and biological activity. These limitations can be addressed by modifying the amyloid fibrils with polyphenols. In this study, two types of polyphenols—tannic acid (TA) and gallic acid (GA)—were selected to prepare hydrogels with soy protein fibrils (SPIFs) at varying proportions to investigate structure, gel properties, and biological activity. TEM results revealed that polyphenols are deposited on the surface of SPIFs by hydrogen bonding and hydrophobic interaction to form hybrid supramolecules. The greater the mass ratio of polyphenols to SPIF, the more pronounced the structural changes. When the mass ratios of TA, GA, and SPIF were 1:20 and 1:2, respectively, the β-sheet content reached the maximum. The gel strength increased by 6 times and 5 times, respectively, with the modulus reaching 334.91 Pa and 317.79 Pa, respectively. The hydrogels exhibited optimal apparent viscosity and structural recovery properties. Bacteriostatic and cytotoxicity tests demonstrated that the hydrogels exhibited excellent antibacterial properties while maintaining safety. In summary, TA demonstrates significant advantages in remodeling SPIF at low concentrations, thereby enhancing the gel characteristics and antibacterial properties of the hydrogel.

## 1. Introduction

In recent years, sustainable development has emerged as a global objective, with plant-derived foods recognized as a significant trend for future advancement. Among these, plant protein has attracted considerable attention from researchers as a promising and sustainable alternative to traditional protein sources [1]. Soy protein isolate (SPI), the most prevalent source of plant-based protein, offers several advantages, including high nutritional value, low cost, and sustainability [2]. SPI possesses various functional properties that make it suitable for food applications, such as gelation, emulsification, foaming, and film formation [3]. The gel characteristics of SPI have generated significant interest in both industry and academia, creating opportunities for the development of related products. At present, the market for plant-based proteins is experiencing rapid growth. However, the gelation properties of natural SPI are insufficient to meet consumer functional requirements. These properties can be enhanced through physical, chemical, or enzymatic treatments. The preparation of SPI into soy protein fibrils (SPIF) is an effective method for significantly enhancing gelation and broadening the range of potential applications for SPI [4,5,6].

Protein amyloid fibrils were initially discovered in medical research. Nevertheless, as research advanced, food protein fibrillation has emerged as a processing method that enhances the functional properties of proteins [7]. Compared to natural proteins, amyloid fibrils exhibit a distinct cross β-sheet structure and an extremely high aspect ratio. These characteristics lead to unique interfacial adsorption and rheological behaviors, thereby effectively enhancing the gelation properties of proteins at low concentrations [8]. Food proteins exhibit the capability to form amyloid fibrils, demonstrate a rich and diverse structural polymorphism, and possess the potential to form hydrogels for nutrient delivery applications [9]. Amyloid fibrils demonstrate specific bioactivities, including bacteriostatic properties, which substantially enhance the potential applications of food proteins and broaden its utility. Notably, polyphenols can remodel amyloid fibrils, significantly improving gel properties and enhancing gel bioactivity.

Polyphenols are significant secondary metabolites found in plants, particularly abundant in fruits, tea, and vegetables. These secondary metabolites exhibit superior bacteriostatic and antioxidant properties [10]. In amyloid fibril hydrogels, polyphenols can engage in hydrogen bonding, π–π stacking, and hydrophobic noncovalent interactions, leading to the formation of hybrid supramolecular structures on the surface of amyloid fibrils. The interaction significantly enhances the gel strength, antibacterial activity, thermal stability, and other properties of hydrogels [11]. Research by Ma et al. [12] shows that when the molar ratio of EGCG to β-lactoglobulin is 2.5:1, the elasticity of hydrogel increases 4 times, and the chewiness is increased by 5 times. Furthermore, the gallate groups of polyphenols exert a pronounced effect on the gelation process. Compared to EGCG, the impact of EGC on amyloid fibrils is significantly diminished when the gallate group is removed. Studies have shown that the gallate group plays a crucial role in inhibiting fibril formation, promoting fibril depolymerization, and reducing the toxicity of amyloid fibrils. Nie et al. [13] and Zhan et al. [14] demonstrated that gallate groups in ECG or EGCG can promote the depolymerization of Aβ protofilaments with amyloid fibrils. Currently, research primarily concentrates on the impact of tea polyphenols with gallate groups on the structural and functional attributes of amyloid fibrils, including their gel forming [12], emulsifying [15], and antibacterial properties [16]. These characteristics are utilized for nutrient delivery [9], food preservation [17] and texture enhancement [18]. Other polyphenols with gallate groups also exhibit significant potential in remodeling amyloid fibrils and possess favorable biological activities. However, there is a paucity of studies specifically addressing the structural and functional properties of food amyloid fibrils, in particular to the application in the food field. This study aims to investigate the potential of polyphenols with gallate groups in remodeling the structure of amyloid fibrils and enhancing their gel properties. Additionally, it will examine their biological activities, which can contribute to expanding their application potential in the food industry.

To explore the effects of other polyphenols with gallate groups on the remodeling of food amyloid fibril structure and gel properties, in this study, two types of polyphenols, tannic acid (TA) and gallic acid (GA), were selected to compare their effects on the structure and gel properties of SPIF at various ratios: 0, 1:50, 1:30, 1:20, 1:10, 1:5, 1:2, and 1:1. This approach will significantly enhance the food texture and nutrient delivery efficiency, thereby expanding the application potential of other polyphenols containing gallate groups in the food industry. ThT fluorescence and TEM were employed to confirm the successful preparation of SPIF and to observe the morphological changes in polyphenols on SPIF. The interaction mechanisms and structural changes were analyzed using ITC, spectroscopy, and other techniques. After preparing the hydrogel, the structural characteristics, moisture distribution, gel strength, and rheological properties were investigated. Furthermore, the antibacterial efficacy and cytotoxicity of the hydrogel were examined. This study elucidates the interaction mechanism between polyphenols and amyloid fibrils, offering a novel perspective for investigating amyloid fibril–polyphenol hydrogels.

## 2. Materials and Methods

### 2.1. Materials

Skimmed soybean flour was procured from Yihai Jiali Cereals, Oils and Foodstuffs Industry Co., Ltd. (Harbin, China). Gallic acid (purity ≥ 98.5%), tannic acid (purity ≥ 96%), and other analytical-grade reagents were purchased from Yuanye Bio-Technology Co., Ltd. (Shanghai, China).

### 2.2. SPI Extraction

The extraction of SPI followed on the method of Wang et al. [19] with slight modifications. The process began by dissolving defatted soybean powder in deionized water and adjusting the pH to 8.0, which was maintained for 2 h. The mixture was centrifuged at 10,000 rpm for 30 min at 4 °C to obtain the supernatant. The supernatant pH was then adjusted to 4.5 and held constant, followed by centrifugation at 6000 rpm for 30 min at 4 °C to collect the precipitate. The precipitate was washed thoroughly with deionized water and centrifuged twice at 6000 rpm for 10 min each at 4 °C. Finally, the pH was adjusted to 7.0, and the product was freeze-dried. The protein content, determined using the Kjeldahl nitrogen method, was 92.8 ± 2.2%.

### 2.3. Preparation of SPIF

Preparation of soy protein fibrils was conducted according to the method of Li et al. [18] with some modifications. Dissolved 5 g of SPI in 100 mL of deionized water to prepare a 5% (*w*/*v*) protein solution. Subsequently, added 2 M hydrochloric acid to adjust the pH to 2 and allowed the solution to hydrate overnight. The sample was processed by centrifugation at 10,000 rpm (11,180× *g*) for 30 min at a temperature of 4 °C to eliminate any insoluble materials. According to the Kjeldahl nitrogen determination method [20], the protein content was determined. The supernatant after centrifugation was heated with concentrated sulfuric acid and catalyst to convert organic nitrogen into inorganic nitrogen, and then the protein content was directly measured using an automatic Kjeldahl nitrogen determination instrument (FoodALYT D4000 Kjeldahl analyzer (OMNILAB GmbH & Co. KG, Bremen, Germany)). The protein content, as determined via the Kjeldahl analysis, was found to be 39.7 ± 0.07 mg/mL. To achieve a final concentration of 35 mg/mL, additional hydrochloric acid with pH2 was added. Finally, the solution was heated at 85 °C with continuous stirring at 200 rpm (4.5 g) for 24 h to obtain SPIF.

### 2.4. Preparation of SPIF-TA/SPIF-GA Complexes

Dissolved TA/GA into solutions at varying concentrations, and subsequently combined them with the SPIF solution [21]. At room temperature, the polyphenol solution and SPIF solution were stirred for 3 h in a dark and oxygen-free environment to keep the two components fully mixed. The mass ratios of TA/GA to SPIF were set at 0, 1:50, 1:30, 1:20, 1:10, 1:5, 1:2, and 1:1. These mixtures were designated as SPIF, F-TA_50_/F-GA_50_, F-TA_30_/F-GA_30_, F-TA_20_/F-GA_20_, F-TA_10_/F-GA_10_, F-TA_5_/F-GA_5_, F-TA_2_/F-GA_2_, and F-TA_1_/F-GA_1_, respectively.

#### 2.4.1. Determination of ThT Fluorescence Intensity

Following the approach outlined by Mao et al. [16], the addition of NaCl increases the concentration of NaCl in both the phosphate buffer (10 mM, pH 7.0) and the ThT stock solution (0.8 mg/mL) to a final concentration of 150 mM. During the determination process, the ThT stock solution was diluted 50 times and mixed with the sample to be analyzed. Measurements were taken using a spectrophotometer (RF6000; Hitachi Ltd., Tokyo, Japan) at an excitation wavelength (λex) of 440 nm and an emission wavelength (λem) of 482 nm.

#### 2.4.2. Transmission Electron Microscopy (TEM) and Sample Appearance

Dilution of SPIF/SPIF–polyphenol complex to a concentration of 0.05 mg/mL for negative staining [15]. Subsequently, 10 μL of the solution was carefully placed on a carbon-coated copper grid. After an incubation period of 2 min, excess solution was wicked away using filter paper. The sample was then air-dried in a desiccator at 25 °C. Following this, the samples were stained with a 10 mg/mL solution of phosphotungstic acid for 2 min. The microstructure of the samples was captured using a Hitachi HT7800 transmission electron microscope (Hitachi High-Tech Company, Tokyo, Japan) operating at an accelerating voltage of 80 kV.

#### 2.4.3. Isothermal Titration Calorimetry (ITC)

The determination was conducted at 25 °C using an isothermal titrator (MicroCal PEAQ-ITC (Malvern Panalytical Inc., San Jose, CA, USA)) [22]. The SPIF solution (pH 2.5) was placed in the reaction chamber of the instrument as the titrant. Subsequently, in two separate experiments, TA and GA polyphenol solutions (pH 2.5) were injected into the reaction chamber using a syringe. The following experimental parameters were established: a total of 19 injections were conducted, with a constant rotation speed of 750 rpm during titration, an injection interval set at 120 s, and each injection lasting exactly 4 s. The polyphenols were titrated into deionized water as a control.

#### 2.4.4. Fourier Transform Infrared Spectroscopy (FTIR)

According to the method of Yao et al. [23] with minor modifications, SPIF and SPIF–polyphenol complexes were freeze-dried, ground with potassium bromide, and subsequently tableted. Potassium bromide was used as a blank for background scanning. The samples were analyzed using a Nicolet iS50 Fourier-transform infrared (FTIR) spectrometer (Thermo Fisher Scientific, Waltham, MA, USA). The scanning range was set from 4000 to 400 cm^−1^, with a total of 32 scans. The spectrum in the region of 1600 to 1700 cm^−1^ was analyzed using OMNIC 8.0 (Thermo Fisher Scientific, Waltham, MA, USA) and Peakfit V4.12 (Sea Solve Software Inc., Framingham, MA, USA). Baseline correction was performed first, followed by Gaussian deconvolution and fitting using the second derivative. Consequently, the ratio of the secondary structure components was calculated based on the area of each sub-peak.

#### 2.4.5. Ultraviolet Spectra Measurement (UV)

SPIF/SPIF–polyphenol complexes were diluted to a concentration of 0.1 mg/mL, and the UV–Vis spectrum was measured in the range of 200–500 nm using a UV spectrophotometer (Shimadzu, Tokyo, Japan) [24].

#### 2.4.6. Fluorescence Spectroscopy Measurement

Diluting the SPIF/SPIF–polyphenol complex solution to 0.1 mg/mL, the λex was set to 280 nm, and the λem in the range of 300–500 nm was collected [25].

#### 2.4.7. X-Ray Diffraction (XRD)

The XRD analysis of the sample was conducted to collect data for 2θ angles ranging from 5° to 50° using a diffractometer (MM007HF/R-AXIS RAPID II, Rigaku Industrial Corporation, Osaka, Japan). The instrument operated at a voltage of 40 kV and a current of 40 mA [26].

#### 2.4.8. Surface Hydrophobicity (H_0_)

According to the method developed by Kato and Nakai [27], minor modifications were implemented prior to conducting the determination. Diluted sample solutions of various concentrations were mixed with 50 μL of an 8 mM ANS solution and allowed to react in the dark for 15 min. The fluorescence intensity data were then measured, with λex set at 390 nm and λem set at 470 nm.

#### 2.4.9. Free Sulfhydryl (−SH) Content

The SPIF/SPIF–polyphenol complex was dispersed in a Tris-Gly buffer (comprising 0.086 mol/L Tris, 0.09 mol/L glycine, and 4 mmol/L EDTA at pH 8.0) at a concentration of 8.0 mg/mL. After centrifugation at 10,000 rpm for 10 min, 5 mL of the solution was taken and mixed with 50 μL of Ellman’s reagent by shaking well. The absorbance was measured at 412 nm following 30 min of stringent light protection [28]. The following formula was used to calculate −SH contents:

(1) $$-SH(\mu mol/g)=\frac{73.35\times A\times D}{C},$$

where A denoted the absorbance of the sample at 420 nm, D indicated the dilution factor, and C represented the protein concentration in mg/mL.

### 2.5. Preparation of Hydrogel

Heated the SPIF/SPIF–polyphenol complex solution in a water bath maintained at 85 °C for a duration of five minutes, followed by rapid cooling in an ice-water bath, resulting in the formation of a hydrogel; among them, F-T_5_, F-T_2_, and F-T_1_ delaminated and could not form hydrogels [29].

#### 2.5.1. Determination of Hydrogel Texture (TPA)

The gel strength was measured using a texture analyzer (TA, XT Plus C, Stable Micro Systems, Stable Micro Systems, Godalming, UK). The probe used was P/0.5, and the test speed was set at 1.0 mm/s [30].

#### 2.5.2. Water-Holding Capacity (WHC)

According to the method described by Ji et al. [9], with slight modifications, 2 g of hydrogel was placed into a 10 mL centrifuge tube and centrifuged at 3000 rpm for 30 min. The surface moisture of the gel was then dried, and the sample was weighed. The water-holding capacity (WHC) was calculated using the following formula:

(2) $$WHC(\%)=\frac{m_{2}-m_{0}}{m_{1}-m_{0}}$$

where m_0_, m_1_, and m_2_ represented the mass of the centrifuge tube, the mass of the gel and centrifuge tube before centrifugation, and the mass of the gel and centrifuge tube after centrifugation, respectively.

#### 2.5.3. Scanning Electron Microscopy (SEM)

According to the method of Wang et al. [31], the SPIF and SPIF–polyphenol hydrogels were freeze-dried and sectioned into small cubes (3 × 3 × 3 mm^3^). These samples were subsequently affixed to a conductive substrate using adhesive tape and sputter-coated with gold. Using EM-30+SEM (COXEM, Daejeon, Korea), the accelerating voltage is 5 kV, and the magnification is ×1K. The images were obtained using XT Microscope Control software (Advanced Analysis Suite v2.5, Stable Micro Systems Ltd.).

#### 2.5.4. Low-Field Nuclear Magnetic Resonance (LF-NMR)

An NMR image analyzer (MesoMR23-060H-I NMR, Suzhou Newmag Analytical Instrument Co., Ltd., Suzhou, China) was employed to determine the water distribution via T_2_ relaxation time. The hydrogel sample was positioned in a cylindrical glass tube with a 15 mm diameter. The transverse relaxation time (T2) was measured using the CPMG pulse sequence. The experimental parameters were set as follows: sampling frequency of 200 kHz, resonance frequency of 21 MHz, echo time of 0.4 ms, waiting time of 3500 ms, and RF delay of 0.002 ms. Data acquisition involved repeating the scan 8 times and collecting 15,000 echoes. [30].

#### 2.5.5. Rheological Properties

A modular rotary rheometer (Thermo Fisher Scientific Shier Technology Co., Ltd., Shanghai, China) equipped with a 35 mm parallel plate configuration (gap = 1 mm) and a P35/Ti probe was utilized to assess the rheological properties of the hydrogel.

##### Frequency Sweep

Prior to testing, the surface of the hydrogel was carefully wiped to remove any excess water, and the sample was placed on a parallel plate rheometer at room temperature (25 °C) for evaluation. The storage modulus (G′) and loss modulus (G″) were recorded over a frequency range of 0.1–10 Hz, and corresponding scanning curves were generated. A control strain of 1% was applied throughout the test [32].

##### Apparent Viscosity

The apparent viscosity (η) was measured at 25 °C with a shear rate ranging from 1 to 100 s^−1^. Then, power law fitting was performed to obtain the values of the consistency index (k) and the flow index (n) [33].

##### Creep-Recovery Experiment

During the initial 100 s, the sample was subjected to a constant stress of 10 Pa to evaluate its strain response. Following this, the stress was removed, and the strain recovery was measured over the subsequent 200 s. The Burgers model was employed to obtain data on the maximum shear strain (γmax), instantaneous modulus of elasticity (G, Pa), instantaneous shear compliance (Je, 1/Pa), and zero-shear viscosity (η, Pa·s) [34].

#### 2.5.6. Antibacterial Activity

The antibacterial activity of SPIF and SPIF–polyphenol hydrogels against *Staphylococcus aureus* (*S. aureus*) was evaluated using the inhibition zone assay [34]. SPIF, TA, and GA served as controls. Bacterial suspensions at a concentration of 1 × 10^6^ CFU/mL were spread on agar plates. Subsequently, 150 μL of hydrogel samples were introduced into Oxford cups, and the zone of inhibition was measured.

#### 2.5.7. In Vitro Cytotoxicity Assay

Cytotoxicity was assessed using the CCK-8 assay. First, 100 µL (1 × 10^3^ to 3 × 10^3^) of human normal colon epithelial cells (NCM460) were added into a 96-well plate and incubated for 24 h. Subsequently, varying concentrations of SPIF or SPIF–polyphenol hydrogel samples were added to the experimental groups, while the control group was treated with a culture medium alone. Each group was tested in parallel for 8 repetitions. Following a 4 h incubation with 10 µL of CCK-8 reagent added to each well, absorbance was measured at 450 nm using a microplate reader, which indirectly indicated the number of viable cells. The data obtained were normalized [35].

### 2.6. Statistical Analysis

Each experiment was performed a minimum of three times, with the outcomes expressed as the mean ± standard deviation. Statistical significance was assessed using a one-way ANOVA followed by a Duncan’s multiple range test, conducted with SPSS version 27 (SPSS Inc., Chicago, IL, USA).

## 3. Results

### 3.1. ThT Fluorescence Intensity Analysis

**Figure 1:**
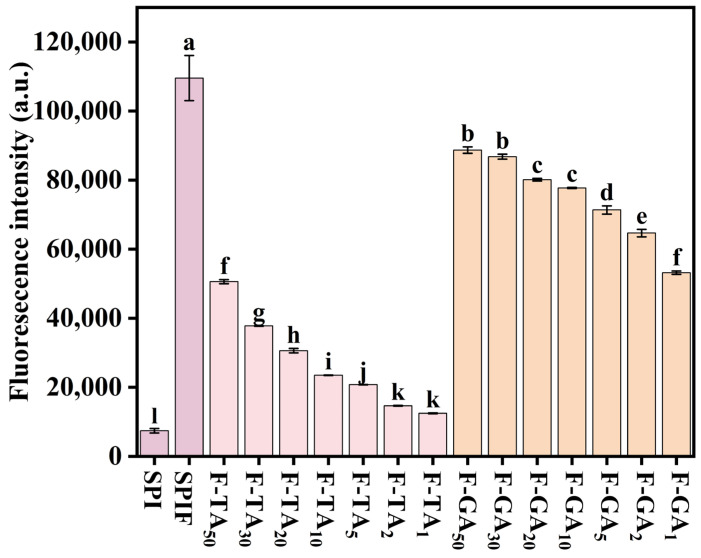
ThT fluorescence intensity of SPI, SPIF, SPIF-TA, and SPIF-GA complexes. The different superscript letters indicate significant differences (*p* < 0.05). SPI: soy protein. SPIF: soy protein fibril. TA: tannic acid. GA: gallic acid.

ThT specifically binds to β-sheet structures and exhibits remarkable specificity for amyloid fibrils [36]. The ThT fluorescence results for SPI, SPIF, and SPIF–polyphenol complexes are presented in Figure 1; compared to SPI, the treated SPIF demonstrated the highest fluorescence intensity, which could be attributed to a greater abundance of β-sheet structures in the prepared SPIF. This observation confirmed the successful preparation of SPIF. The addition of TA or GA resulted in a decrease in fluorescence intensity. The β-sheet structure in amyloid fibrils was primarily stabilized by hydrophobic interactions, π–π stacking of aromatic amino acids, and hydrogen bonds [16]. The incorporation of polyphenols could disrupt the β-sheet structure of SPIF through various non-covalent interactions. Some studies also indicated that polyphenols compete with ThT for binding sites on protein fibrils, exhibiting dose-dependent inhibition and thereby altering the fluorescence intensity results [37]. Compared to GA, TA led to a more rapid decline in fluorescence intensity. This phenomenon could be attributed to the stronger affinity between TA and SPIF, which could facilitate stronger noncovalent interactions, such as hydrogen bonding, leading to a more significant reduction in fluorescence intensity. In summary, protein fibrils were successfully synthesized via acid-heat treatment of soy protein. The addition of polyphenols led to a reduction in ThT fluorescence intensity, likely due to the disruption and interference with the β-sheet structure of the fibrils by polyphenols.

### 3.2. TEM and Sample Appearance Analysis

**Figure 2:**
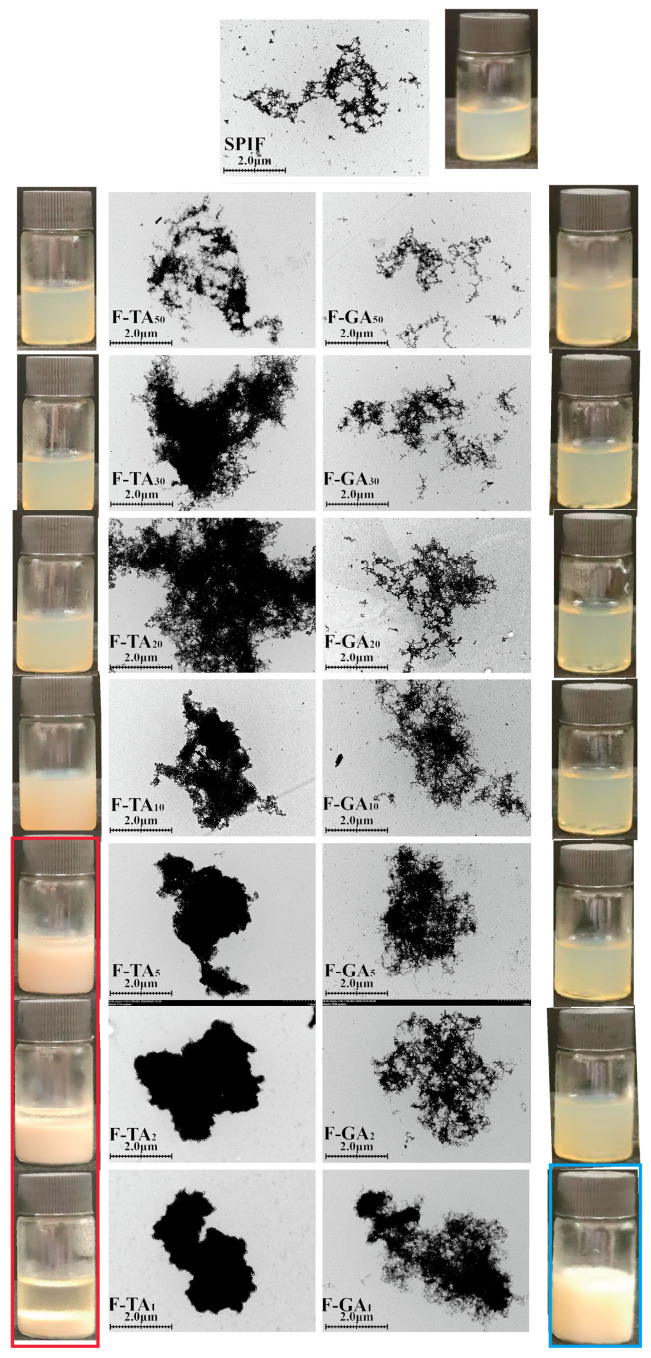
TEM images alongside sample images of SPIF, SPIF-TA, and SPIF-GA complexes. SPIF: soy protein fibril. TA: tannic acid. GA: gallic acid.

The structural characteristics of protein fibrils and the impact of polyphenols on the micro-morphology of SPIF can be effectively observed using TEM. As illustrated in Figure 2, SPIF exhibited a long, branched fibril structure with a high aspect ratio, indicating the successful preparation of soy protein fibrils. The addition of TA or GA significantly altered the structure of SPIF. Within a specific concentration range, the incorporation of TA led to a denser protein fibrils structure. This phenomenon occurred due to the accumulation and development of polyphenols on the surface of SPIF, leading to the formation of polyphenol–amyloid hybrid nanofilaments [38]. However, when the mass ratio of TA to SPIF reached 10:1, TA compromised the structural integrity of SPIF, resulting in the formation of a denser fibril cluster. At ratios of 5:1 or higher, the fibrillar structure of SPIF was completely disrupted, leading to the formation of more compact aggregates. The findings were attributed to the high concentration of gallate groups in TA, which could have both hydrophobic and hydrogen-bonding interactions with SPIF. The elevated polyphenol concentrations might facilitate the transformation of SPIF into non-amyloid fibril aggregates. Meanwhile, studies have shown that EGCG is capable of transforming large, mature α-synuclein and amyloid-β fibrils into smaller, non-toxic, amorphous protein aggregates that were harmless to mammalian cells [39], and TA could exert an effect on SPIF that is similar to that of EGCG.

In contrast, GA at a low mass ratio had a minimal impact on the structure of the SPIF. With the increase in polyphenol proportion, GA was gradually adsorbed onto the surface of SPIF, leading to the accumulation and formation of polyphenol–amyloid hybrid nanofilaments. The structural modifications essentially ceased when the mass ratio reached 2:1. At a mass ratio of 1:1, certain regions of the hybrid nanofilaments exhibited excessive aggregation. Previous studies have demonstrated that GA can effectively inhibit the formation of insulin amyloid fibrils [40]. However, recent findings indicated that its effect on pre-formed fibrils was limited. The above results indicated that the influence of GA on the structure of SPIF was less pronounced compared to that of TA. It showed that polyphenols can significantly change the micro-morphology of SPIF; in contrast, TA effect was more significant.

From the observations, with the incremental addition of TA, the sample exhibited a homogeneous solution with a progressively deepening color. But when the mass ratio reached 5:1 or higher, sedimentation occurred, and the supernatant became increasingly pronounced (red box). This phenomenon was attributed to the formation of amorphous protein aggregates. Conversely, the addition of GA had minimal impact on the color. At a mass ratio of 1:1, the color shifted to white (blue box), likely due to an excessive addition of polyphenols.

### 3.3. ITC Analysis

**Figure 3:**
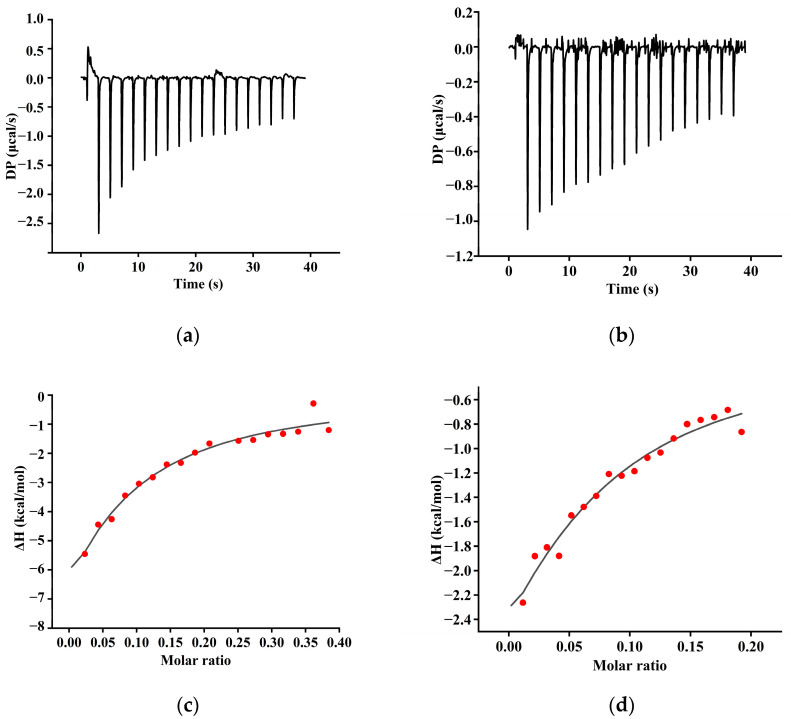
Isothermal titration calorimetry (ITC)-binding isotherms for the interaction between SPI and natural polyphenol (TA (**a**), GA (**b**)). Calorimetric curves (T = 298 K) of titration. Fit curves of the sequential binding model of (TA (**c**), GA (**d**)). SPIF: soy protein fibril. TA: tannic acid. GA: gallic acid.

The enthalpy changes associated with polyphenol–protein interactions can be accurately quantified using ITC, thereby enabling the precise determination of thermodynamic parameters between different polyphenols and proteins (Figure 3). The continuous exothermic response observed during the titration of the TA or GA indicated that the interactions between these polyphenols and SPIF were primarily enthalpy-driven [41]. The titration of the two polyphenols resulted in a gradual leveling off of the heat of reaction, suggesting that the binding sites on SPIF become increasingly saturated. The binding constants (Kd) for the TA or GA with SPIF were as follows: SPIF-TA (2.39) < SPIF-GA (27.8). This indicated that TA exhibits stronger non-covalent interactions with SPIF, likely due to its unique structure, which features a central glucose molecule surrounded by ten gallate groups. These gallate groups could interact with proteins through hydrogen bonding, hydrophobic interactions, and electrostatic forces, resulting in a higher affinity for SPIF [42].

According to Table 1, ΔG < 0 and ΔH < 0, indicating that the spontaneous exothermic reaction between the two polyphenols (TA and GA) and SPIF were enthalpy-dominated. Furthermore, based on the Gibbs energy equation ΔG = ΔH − TΔS, with ΔH < 0 and ΔS < 0, it could be speculated that the interaction between the TA and GA and SPIF was primarily driven by hydrogen bonding and hydrophobic interactions [43]. For TA, |ΔH| > |−TΔS|, suggesting that the predominant force between TA and SPIF was hydrogen bonding. Additionally, the Gibbs free energy changes ΔG: GA (−3.49) > TA (−5.72), suggesting that the interaction between SPIF and TA was more thermodynamically favorable. Overall, these results indicated that the interaction between the two polyphenols and SPIF was spontaneous, exothermic, and primarily driven by hydrogen bonding and hydrophobic interactions.

### 3.4. FTIR Analysis

**Figure 4:**
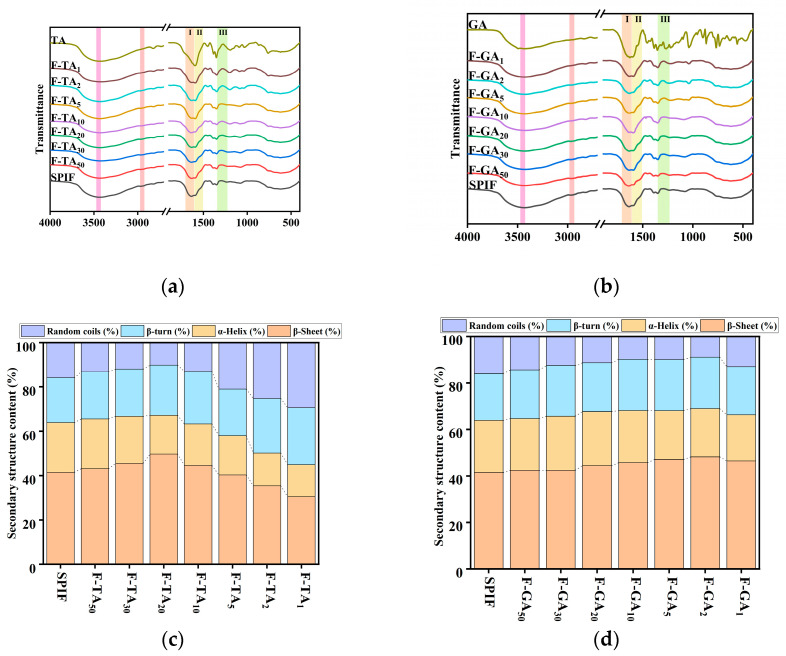
FTIR images of SPIF, TA, and SPIF-TA complexes (**a**) and SPIF, GA, and SPIF-GA complexes (**b**). Secondary structure content of SPIF and SPIF-TA complexes (**c**) and SPIF and SPIF-GA complexes (**d**). SPIF: soy protein fibril. TA: tannic acid. GA: gallic acid. I: amide I band; II: amide II band; III: amide III band.

As shown in Figure 4a,b, TA or GA exhibited a distinct characteristic peak. However, upon combination with SPIF, this characteristic peak vanished, suggesting that an interaction between the two components had indeed taken place. The long and broad peak at 3433 cm^−1^ was shifted (TA: 3433 to 3420 cm^−1^, GA: 3433 to 3427 cm^−1^), suggesting that hydrogen bonding played a significant role in the interaction between TA/GA and SPIF [44]. Specific analysis indicated that the prominent and broad characteristic peak (amide A) in SPIF was primarily attributed to the stretching vibrations of the O-H and N-H groups. Additionally, the characteristic amide B peak near 2960 cm^−1^ exhibited a noticeable red shift and gradually became broader with the addition of polyphenols, suggesting that hydrophobic interactions were also involved. Meanwhile, both the amide II and amide III bands, which were attributed to N-H and C-N bending vibrations [45], also changed the peak intensity upon the addition of polyphenols.

As shown in Figure 4c,d, acid heat treatment led to a significant increase in the β-sheet content, attributed to the enhancement of both parallel and antiparallel β-sheet structures after the formation of SPIF. At first, the β-sheet content increased, which aligned with the findings of Mao et al. [16] in the circular dichroism studies. It was speculated that the effect may be due to the addition of a small amounts of polyphenols, which could strengthen the structure of SPIF and enhance its structural order. When the mass ratio of TA to SPIF reached 20:1 or the mass ratio of GA to SPIF reached 2:1, the β-sheet content reached its maximum values of 49.65% (for F-TA_20_) and 48.19% (for F-GA_2_), respectively. However, when this ratio was mass ratio, the proportion of random coils exhibited a substantial increase. This occurred because polyphenols diminish the stability of SPIF, reduce the number of hydrogen bonds between chains, and convert β-sheet into random coil structures. The results indicated that there are two types of noncovalent interactions between TA and GA with SPIF. A low concentration of polyphenols increased the β-sheet structure of SPIF, whereas a high concentration led to a substantial rise in the random coil content. Notably, TA exerted a more pronounced effect on the secondary structure compared to GA.

### 3.5. UV Analysis

**Figure 5:**
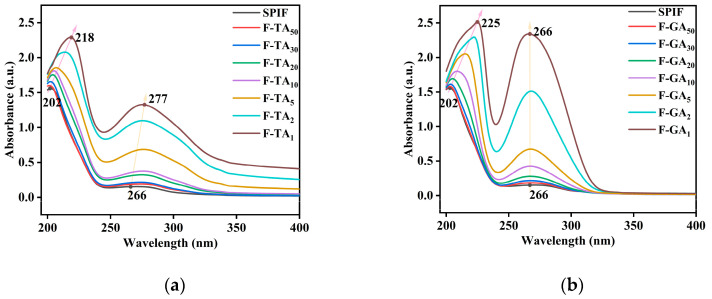
UV spectra of SPIF, SPIF-TA complexes (**a**) and SPIF, SPIF-GA complexes (**b**). SPIF: soy protein fibril. TA: tannic acid. GA: gallic acid.

The UV spectrum is depicted in Figure 5, where SPIF exhibited two absorption peaks at 204 nm (the larger peak) and 266 nm (the smaller peak). The absorbance increased with higher mass ratio of both GA and TA. Notably, the first absorption peak exhibited a red shift upon the addition of TA, shifting from 202 nm to 218 nm, and GA, shifting from 202 nm to 225 nm. In contrast, the second absorption peak showed a red shift only with TA (from 266 nm to 277 nm), while GA did not induce any significant change (remaining at 266 nm). This indicated that TA altered the microenvironment surrounding aromatic amino acids to a greater extent than GA. However, GA resulted in a greater change in peak intensity, the relatively low molecular weight of GA facilitated the formation of a stronger conjugated system near these aromatic amino acids. As a result, the energy of the π–π* transition increased, leading to the formation of a new complex with an extinction coefficient higher than that of the protein itself [46]. However, TA could not be closely associated with aromatic amino acids in SPIF due to steric hindrance. Polyphenol enhanced the two absorption peaks of SPIF, while GA more intensified the absorption peaks due to its molecular weight.

### 3.6. Fluorescence Spectroscopy Analysis

**Figure 6:**
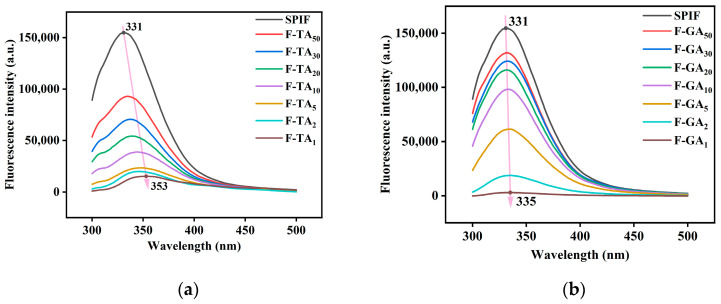
Fluorescence spectra of SPIF, SPIF-TA complexes (**a**) and SPIF, SPIF-GA complexes (**b**). SPIF: soy protein fibril. TA: tannic acid. GA: gallic acid.

As illustrated in Figure 6, the maximum fluorescence intensity of SPIF occurred at 331 nm. Upon the addition of TA/GA, the fluorescence intensity decreased continuously in a dose-dependent manner, as polyphenols interact with aromatic amino acids to form non-luminescent ground state complexes [45]. Compared to GA, the incorporation of TA led to a faster reduction in fluorescence intensity. However, when the mass ratio of polyphenols to SPIF reached 1:1, GA caused the fluorescence intensity to reach its lowest point. This phenomenon could be attributed to the steric hindrance associated with the large molecular weight of TA. In addition, the incorporation of polyphenols induced a redshift in the maximum fluorescence intensity (TA: 331 nm to 353 nm, GA: 331 nm to 335 nm). This shift suggests a conformational change in the surrounding environment of the aromatic amino acids, leading to structural unfolding and exposure to a more hydrophilic and polar environment.

### 3.7. XRD Analysis

**Figure 7:**
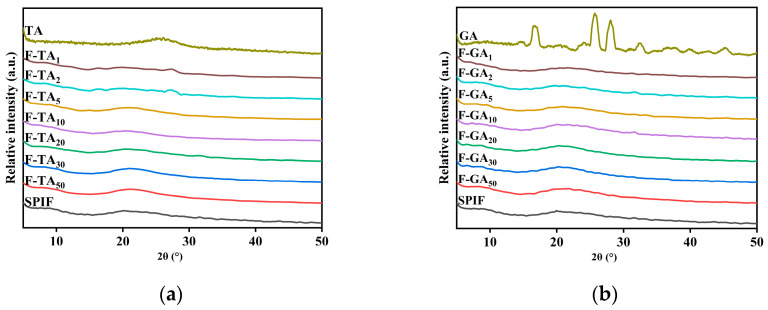
XRD images of SPIF, SPIF-TA complexes (**a**) and SPIF, SPIF-GA complexes (**b**). SPIF: soy protein fibril. TA: tannic acid. GA: gallic acid.

Cross β-sheet structures can exhibit distinct X-ray diffraction patterns. As illustrated in Figure 7, SPIF displayed two broad, flat peaks at 8.7° and 19.4°, consistent with the findings of Ji et al. [9]. It demonstrated the successful preparation of SPIF. TA and GA had distinct structural characteristics. After the addition of polyphenols to SPIF, the distinct peak associated with polyphenols vanished. This observation indicated that polyphenols interacted with SPIF rather than merely being mixed together. Within a certain range, the addition of TA/GA had minimal impact on the peak value of SPIF. However, when excess polyphenols were added, the intensity of the characteristic peaks decreased, with the first characteristic peak nearly disappearing. This phenomenon could be attributed to the competitive binding between TA/GA and the peptide chain of the protein, which resulted in the occupation of binding sites. Consequently, this inhibition prevented the protein from cross-binding and decreased the formation of the cross β-sheet structure [16], indicating that the addition of polyphenols can disrupt the amorphous structure of SPIF and affect the cross β-sheet structure. The above results showed that polyphenols and SPIF will not affect the two amorphous structures of SPIF at low mass ratio, but would destroy the amorphous structures at high mass ratio, and TA’s effect could be obvious.

### 3.8. H_0_ Analysis

**Figure 8:**
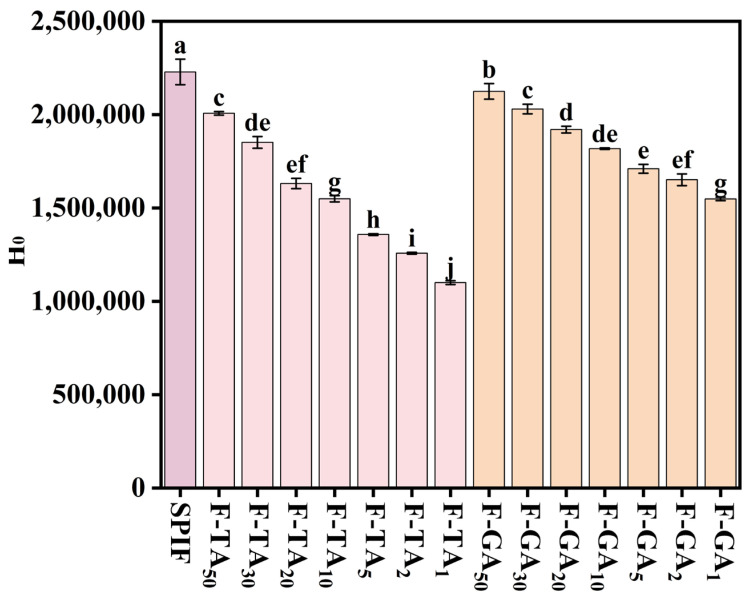
H_0_ of SPIF, SPIF-TA, and SPIF-GA complexes. The different superscript letters indicate significant differences (*p* < 0.05). SPIF: soy protein fibril. TA: tannic acid. GA: gallic acid.

SPIF showed the greatest H_0_, whereas incorporating TA/GA led to a reduction in this property (Figure 8), which could be attributed to the following reasons: (1) Palhano et al. [47] had demonstrated that polyphenols can bind to the hydrophobic sites of amyloid fibrils, thereby remodeling their structures. These findings suggested that TA and GA bind to hydrophobic sites and establish a competitive interaction with ANS. Consequently, the addition of polyphenols resulted in a reduction in hydrophobic groups on the surface, which in turn led to a decrease in H_0_. (2) TA/GA contain a significant number of hydroxyl groups, which could enhance overall hydrophilicity upon complex formation. Furthermore, compared to GA, TA led to a more pronounced decrease in H_0_, likely due to its higher content of hydroxyl groups, which significantly enhanced the hydrophilicity of the complexes. Polyphenols could reduce H_0_ by introducing hydroxyl groups and destroying the hydrophobic structure of SPIF.

### 3.9. −SH Content Analysis

**Figure 9:**
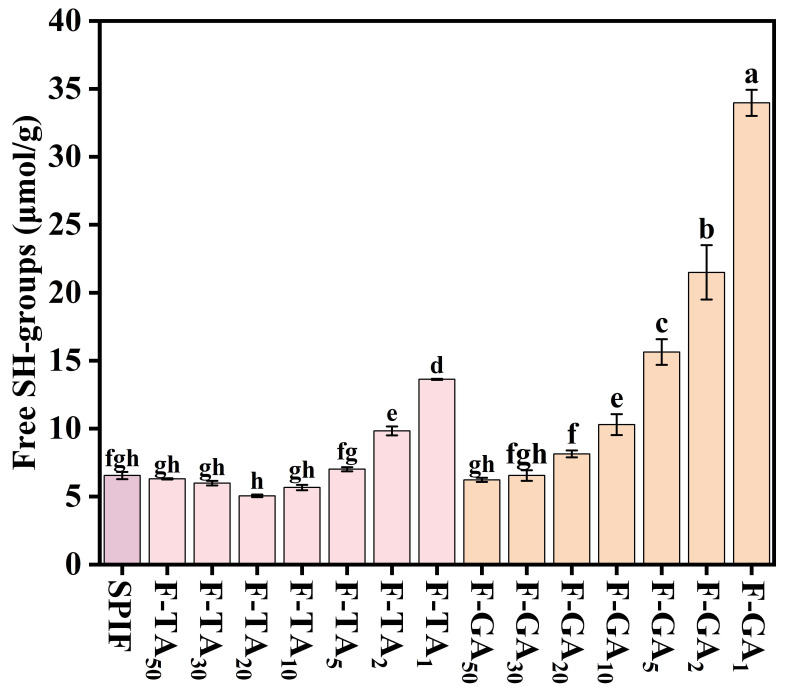
−SH content of SPIF, SPIF-TA, and SPIF-GA complexes. The different superscript letters indicate significant differences (*p* < 0.05). SPIF: soy protein fibril. TA: tannic acid. GA: gallic acid.

Free sulfhydryl groups play an important role in amyloid fibril formation. Yang et al. [48] observed a gradual decrease in −SH content in the process of forming amyloid fibril, which was attributed to the conversion of −SH into disulfide bonds. As illustrated in Figure 9, the −SH content initially decreased slightly with the addition of TA/GA. This reduction was attributed to noncovalent interactions between the −SH in SPIF and the hydroxyl groups present in the polyphenols. Wei et al. [49] also discovered that the non-covalent interactions of various polyphenols, including tannins, quercetin, and resveratrol, with the myofibrillar proteins of *Nemipterus virgatus* resulted in reduced −SH content. Notably, as the polyphenol concentration increased, there was a significant increase in −SH content, which could be attributed to the ability of polyphenols to inhibit fibril formation and disrupt pre-formed fibrils [11]. Both TA and GA promoted the depolymerization of SPIF, leading to the exposure of −SH and the cleavage of certain disulfide bonds. This process ultimately resulted in an increase in −SH content.

### 3.10. Gel Strength and WHC Analysis

**Figure 10:**
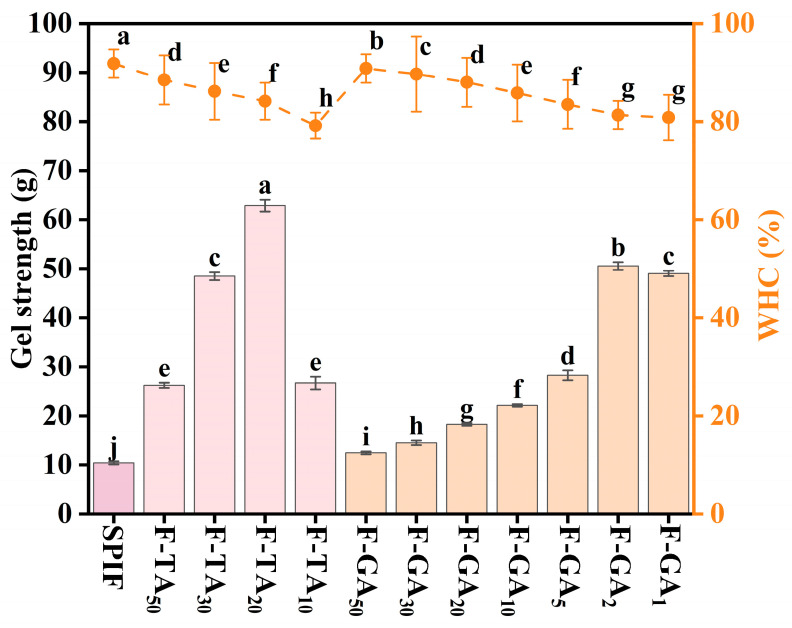
Gel strength (g) and WHC (%) of SPIF, SPIF-TA, and SPIF-GA hydrogels. The different superscript letters indicate significant differences (*p* < 0.05). SPIF: soy protein fibril. TA: tannic acid. GA: gallic acid.

Gel strength effectively reflects the fundamental characteristics of protein gels. As illustrated in Figure 10, the hydrogel formed exclusively by SPIF exhibited notably low strength (10.45 g). When polyphenols were added, the gel strength was significantly enhanced. This improvement can be attributed to the ability of polyphenols to act as effective gelling agents, thereby facilitating the gelation process. According to Zhong et al. [50], puerarin exhibited dual properties, serving both as a gelling agent and as a nutritional health supplement in whey protein heat-induced hydrogels. The deposition of polyphenols on the surface of SPIF led to the formation of polyphenol–amyloid hybrid nanofilaments, thereby enhancing gel strength [38]. Specifically, when the mass ratios of TA:SPIF and GA:SPIF reached 20:1 and 2:1, respectively, the gel strength increased to more than 6 times and 5 times. However, excessive polyphenols led to the formation of amorphous aggregates of SPIF, reducing the degree of cross-linking and consequently decreasing gel strength.

WHC represents the capacity of the gel network to retain water and is a crucial indicator of gel quality. It was observed that the WHC of the gel decreased with an increasing polyphenol to SPIF mass ratio, when F-TA_10_ and F-GA_1_ were used, the WHC was the lowest, likely due to the formation of a denser gel network. This densification caused the gel system to contract, reducing its ability to store water and leading to an increase in free water within the system. Additionally, the presence of polyphenols could promote amyloid fibril aggregation, facilitating water expulsion from the surface. This result aligned with the research findings of Ji et al. [9]. Excessive polyphenols would lead to a reduction in the gel strength of hydrogels, which could also contribute to a decrease in WHC.

### 3.11. SEM Analysis

**Figure 11:**
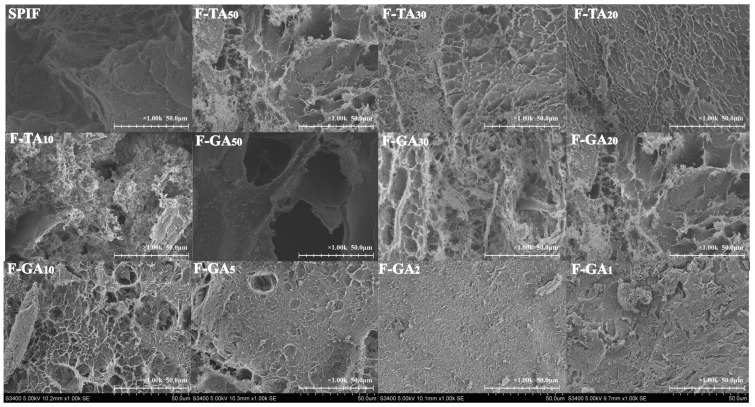
SEM images of SPIF, SPIF-TA, and SPIF-GA hydrogels. SPIF: soy protein fibril. TA: tannic acid. GA: gallic acid.

As evident from Figure 11, the hydrogel formed exclusively by SPIF displayed a notable void defect. This could be attributed to the low protein content, which was insufficient to completely fill the gel matrix. The addition of TA/GA resulted in increased cross-linking among SPIF, leading to a more compact network with smaller pores. Hydrophobic interactions, hydrogen bonding, and π–π stacking interactions were believed to play a significant role in the formation of hydrogels between polyphenols and amyloid fibrils [51]. However, when the mass ratio between TA and SPIF reached 1:10, excessive aggregation of SPIF was observed, leading to a rough and irregular surface that hindered the formation of a uniform and dense network. This alteration in SPIF configuration transformed amyloid fibrils into amorphous aggregates [39], which weakened the interactions between the inter-fibrils. For GA, the gel network density peaked at a ratio of 1:2. However, ratios exceeding 1:1 resulted in a rough gel surface, which could be attributed to an excess of GA. SEM results showed that polyphenols can promote the cross-linking of SPIF and reduce the pore structure of hydrogel, thus improving the gel strength.

### 3.12. LF-NMR Analysis

**Figure 12:**
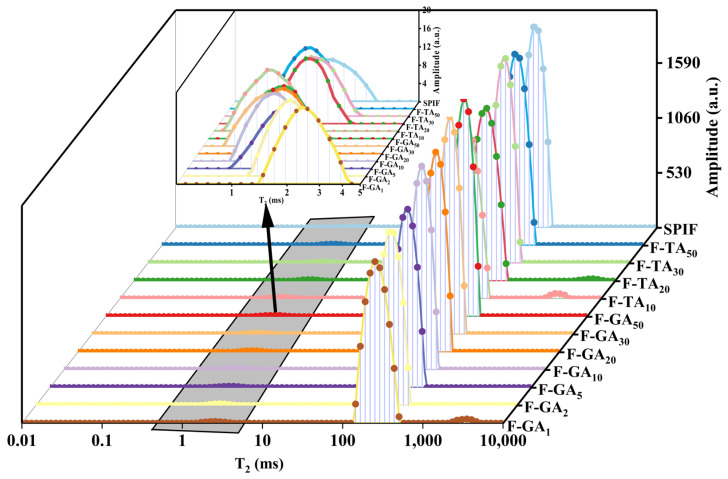
LF-NMR images of SPIF, SPIF-TA, and SPIF-GA hydrogels. SPIF: soy protein fibril. TA: tannic acid. GA: gallic acid.

The relaxation time distribution spectra were presented in Figure 12, and the moisture distribution ratios are summarized in Table 2. It could be observed from the figure that there are two or three distinct peaks in the relaxation time of the hydrogel. The range of 1–10 ms (T_21_) is considered to represent bound water within the gel matrix, while 10–1000 ms (T_22_) indicates immobile water, and 1000–10,000 ms (T_23_) corresponds to free water [52]. The incorporation of polyphenols led to an increase in the proportion of bound water, indicating that polyphenols enhanced the gelation capacity of SPIF hydrogels. The hydrogen bonds present within polyphenols could interact with water molecules, resulting in the transformation of immobile water within the gel into bound water [53]. According to Table 2, the percentage of immobile water in hydrogels with varying polyphenol ratios exceeded 95%, indicating that immobile water was a major constituent of SPIF hydrogels. The addition of polyphenols caused a leftward shift in the still water content, suggesting that polyphenols promote a denser network structure within the gel and reduce pore mobility in the gel. Furthermore, TA resulted in a more significant shift in still water compared to GA, implying that TA enhances the hydrogel network structure to a greater extent [54]. A new peak (1000–10,000 ms) was observed in F-TA_20_, F-TA_10_, and F-GA_1_, indicating the presence of free water in the gel system. This phenomenon could be explained to the contraction of the gel matrix, which was unable to retain the excessive amount of immobile water. For F-TA_10_ and F-GA_1_, this could be linked to a reduction in the gel properties, leading to the conversion of immobile water into free water. These findings explained the observed decrease in the WHC of the hydrogels.

#### 3.12.1. Frequency Sweep Analysis

**Figure 13:**
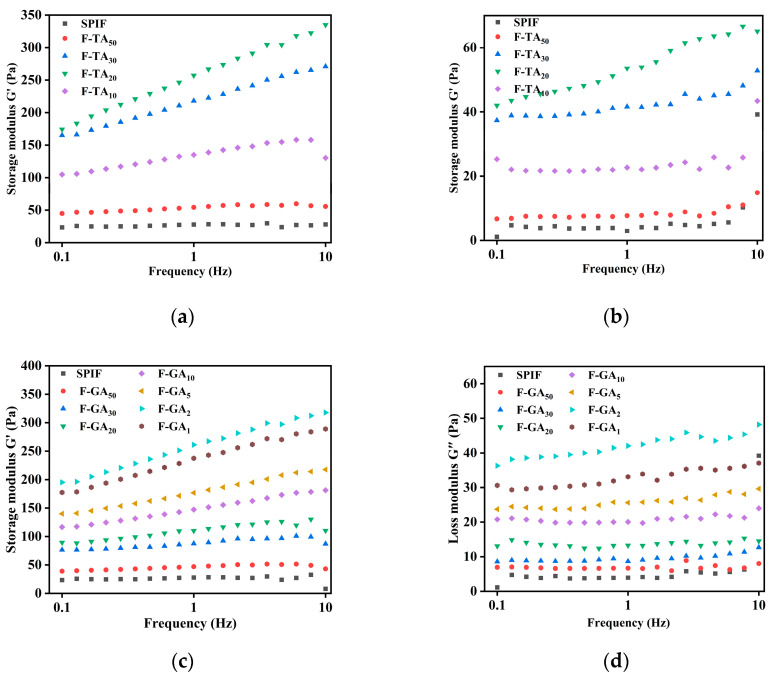
Rheological images of SPIF, SPIF-TA, and SPIF-GA hydrogels. Storage modulus (G′) images of SPIF, SPIF-TA (**a**), and SPIF-GA (**c**) hydrogels by frequency sweep. Loss modulus images (G″) of SPIF, SPIF-TA (**b**), and SPIF-GA (**d**) hydrogels by frequency sweep. SPIF: soy protein fibril. TA: tannic acid. GA: gallic acid.

The frequency-scanning results of the sample at 25 °C across the range of 1–10 Hz were illustrated in Figure 13. The storage modulus (G′) of the hydrogel prepared by SPIF was greater than the loss modulus (G″) at lower frequencies, indicating that tan δ (the ratio of G′ to G″) < 1, demonstrating that the sample exhibited solid characteristics [34]. However, when the frequency reached 10 rad/s, G″ > G′ (tan δ > 1), the hydrogel behaved like a viscous fluid. This led to damage to the gel structure and a reduction in elasticity, indicative of the formation of a relatively weak dissipative viscoelastic gel. The observation aligned with the findings conducted by Tiong et al. [55] on heat-induced gels of pea protein compared to soy protein. With the addition of TA/GA, both G′ and G″ increased significantly, and tan δ < 1 indicated that the gel exhibited solid-like elasticity and low frequency dependence. This suggested the incorporation of polyphenols could enhance the viscosity and elasticity of the hydrogel. Akazawa et al. [56] investigated the effects of cold aqueous extracts of olive leaves (polyphenols) on egg white protein gels. The findings found that the enhancement of gel properties by polyphenols was due to their remarkable protein cross-linking activity, suggesting that TA and GA can improve the elasticity of protein gels through non-covalent interactions. However, excess polyphenols, led to the transformation of SPIF into amorphous aggregates, which exhibited a reduced degree of cross-linking and diminished gel properties.

#### 3.12.2. Apparent Viscosity Analysis

**Figure 14:**
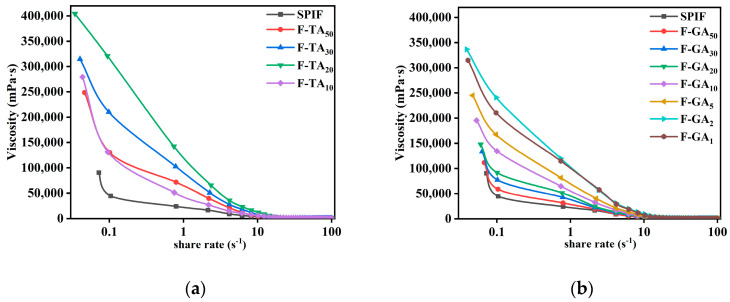
Apparent viscosity images of SPIF, SPIF-TA (**a**), and SPIF-GA (**b**) hydrogels. SPIF: soy protein fibril. TA: tannic acid. GA: gallic acid.

Figure 14 illustrated the apparent viscosity of SPIF and SPIF–polyphenol hydrogels. The apparent viscosity of all hydrogels decreased rapidly with an increase in shear rate (0.1–100 s^−1^), demonstrating pronounced shear-thinning behavior (pseudoplastic fluid). This behavior indicated that the hydrogels produced by SPIF possess a soft texture [57]. At the initial stage, the apparent viscosity decreased rapidly, indicating that the hydrogel exhibited poor resistance to shear stress. The incorporation of polyphenols could significantly improve the viscosity of SPIF hydrogel by acting as cross-linking agents that enhanced the interactions between proteins [56]. In addition, compared to proteins, protein fibrils demonstrated increased apparent viscosity as a result of the development of a dense network structure and an enlargement in the hydrodynamic diameter of the proteins [58]. The incorporation of polyphenols onto the surface of SPIF resulted in the formation of polyphenols–amyloid supramolecules, which exhibited increased disorder in their entanglement and a significant increase in diameter, as demonstrated by TEM results. Consequently, this enhancement significantly increased the apparent viscosity of the hydrogel. But excess polyphenols (F-TA_10_, F-GA_1_) resulted in a decrease in apparent viscosity, likely due to the excessive polyphenols causing protein aggregation and reducing the cross-linking between proteins.

The consistency index (K) and flow index (n) were determined using the power law fitting equation, with R^2^ value > 99% indicating that the fitted model accurately reflected the flow behavior of the samples (Table 3). All samples exhibited a flow index (n) < 1, confirming that they are shear-thinning non-Newtonian fluids [33]. A higher K value corresponded to greater cohesion within the gel. The samples F-GA_2_ (41.61) and F-TA_20_ (49.18) exhibited the highest K values, suggesting that these samples possess the best cohesion at this mass ratio. This finding suggested that incorporating polyphenols increased the gel viscosity and facilitated the development of a strong network structure.

#### 3.12.3. Creep-Recovery Analysis

**Figure 15:**
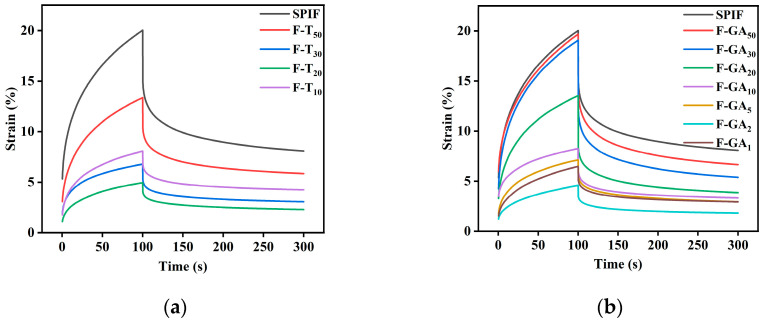
Creep and recovery images of SPIF, SPIF-TA (**a**), and SPIF-GA (**b**) hydrogels. SPIF: soy protein fibril. TA: tannic acid. GA: gallic acid.

As illustrated in Figure 15 and Table 3, all samples exhibited a typical nonlinear response to strain and resilience, indicating that their viscoelastic behavior [59]. In the initial 100 s, the structure of the sample deteriorated under constant stress, leading to a gradual increase in strain. The hydrogel with greater gel strength exhibited a smaller change in strain; specifically, the maximum shear strain (γmax) was reduced. This observation suggested that there may be an inverse proportionality relationship between gel strength and maximum shear strain (γmax) [43]. The instantaneous elastic modulus (G, Pa) increased with higher polyphenol concentrations, Specifically, TA and GA polyphenols enhanced the G of SPIF hydrogel by 4.70 times and 3.86 times, respectively, suggesting that polyphenols facilitate the development of a more tightly and robust network structure via various noncovalent interactions. This enhancement contributed to the increased stiffness and elasticity of hydrogel samples.

Within the latter 200 s, the applied stress was removed, allowing the hydrogel to attempt to return to its original shape. This recovery behavior was influenced by the previously applied stress and the material microstructure. At this stage, the hydrogel structure containing polyphenols exhibited a faster recovery and a shorter relaxation time. This observation suggested that polyphenols enhance the elastic modulus (G′) of the hydrogel [60]. The zero shear viscosity (η) was defined as the viscosity resulting from the entanglement of molecular chains. The incorporation of polyphenols enhanced the cross-linking of proteins, creating additional entanglement sites and thereby improving the stability of the structure. Specifically, F-TA_20_ (8.725) and F-GA_2_ (7.364) showed the highest zero shear viscosity. The higher instantaneous shear flexibility (Je 1/Pa) indicated a reduced ability to maintain the original state, F-TA_20_ (0.3101) and F-GA_2_ (0.3594) exhibited the lowest Je values, indicating that they possessed the highest resistance to degradation. Furthermore, the comparison revealed that TA could markedly improve the creep and recovery properties of the SPIF hydrogel at lower concentrations relative to GA. However, when excessive polyphenols were present, the strain in the samples increased, leading to a decrease in recovery capacity.

### 3.13. Antibacterial Activity Analysis

**Figure 16:**
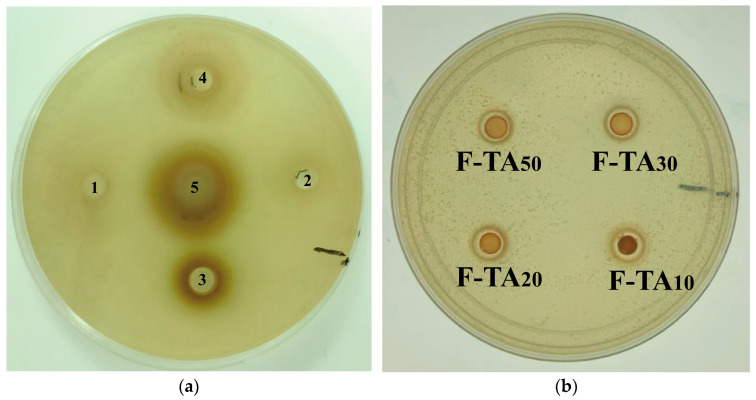

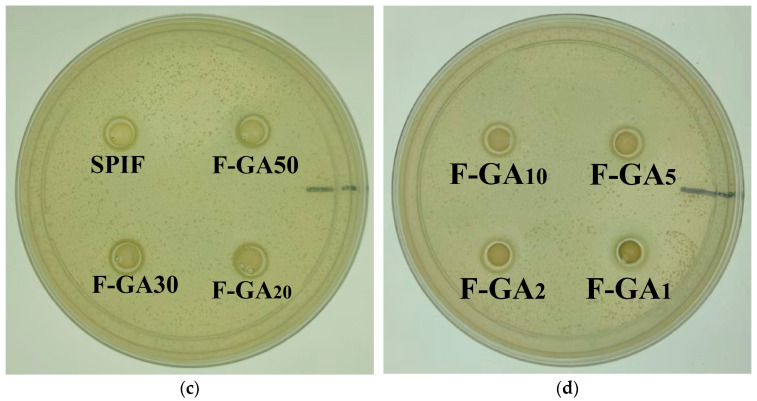
Inhibitory images of TA, GA, SPIF, SPIF-TA, and SPIF-GA hydrogels. (**a**): Bacteriostatic circle of TA and GA. (**b**): The bacteriostatic circle of TA and SPIF at the mass ratio of 1:50, 1:30, 1:20 and 1:10. (**c**): SPIF and the bacteriostatic circle of GA and SPIF at the mass ratio of 1:50, 1:30 and 1:20. (**d**) The bacteriostatic circle of (**d**) GA and SPIF in the mass ratio of 1:10, 1:5, 1:2 and 1:1. Numbers 1, 2, and 3 correspond to the GA contents of SPIF-GA_50_, SPIF-GA_20_, and SPIF-GA_10_, respectively, while numbers 4 and 5 correspond to the TA contents of SPIF-TA_50_ and SPIF-TA_10_, respectively. SPIF: soy protein fibril. TA: tannic acid. GA: gallic acid.

To investigate the bacteriostatic effect of SPIF and SPIF–polyphenol complexes, Gram-positive *Staphylococcus aureus* (*S. aureus*) was selected as the representative microorganism, and the inhibitory effect was evaluated using the inhibition zone method. As shown in Figure 16, the numbers 1, 2, and 3 correspond to the bacteriostatic zones of SPIF and GA at mass ratios of 50:1, 20:1, and 10:1, respectively. The numbers 4 and 5 correspond to the bacteriostatic zones of SPIF and TA at mass ratios of 50:1 and 10:1. Previous studies had demonstrated that SPI alone does not exhibit any bacteriostatic effect on *Staphylococcus aureus* [61]. However, the results indicated that simple SPIF hydrogel exhibits a slight bacteriostatic effect, which aligned with the findings of Xu et al. [4]; this could be attributed to the ability of SPIF to disrupt the bacterial cell membrane, thereby exhibiting its bacteriostatic properties. TA demonstrated effective antimicrobial properties by influencing the expression of proteins involved in bacterial cell wall synthesis or by inhibiting specific enzymes [62]. The incorporation of TA significantly enhanced the bacterial inhibition capacity of the hydrogel. But the antibacterial effect was less pronounced compared to the inhibition zone observed with TA solution alone, likely due to the limited diffusion capability of TA within the hydrogel matrix. The antibacterial efficacy of GA was limited at low concentrations, which is enhanced in hydrogels containing a high concentration. GA might exert its antibacterial effects by inhibiting the formation of bacterial biofilms and extracellular polysaccharides [63]. Finally, the results indicated that the antibacterial effect of the hydrogel can be enhanced through the synergy between SPIF and polyphenols. Among the two polyphenols, TA demonstrated a superior antibacterial effect at lower concentration, highlighting the antibacterial potential of SPIF–polyphenol hydrogels for food applications.

### 3.14. In Vitro Cytotoxicity Analysis

**Figure 17:**
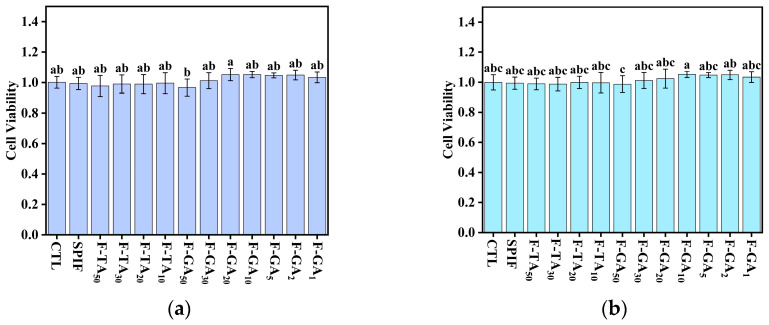
Cytotoxicity images (1.875 (**a**) and 3.75 mg/mL (**b**)) of SPIF, SPIF-TA, and SPIF-GA hydrogels. The different superscript letters indicate significant differences (*p* < 0.05). SPIF: soy protein fibril. TA: tannic acid. GA: gallic acid.

Amyloid fibrils were denatured proteins that were initially discovered in various diseases, raising significant concerns about their safety [64]. Consequently, a CCK-8 proliferation assay was employed to evaluate the inhibitory effects of SPIF and the SPIF–polyphenol complexes on the growth activity of human normal colon epithelial cells (NCM460) and to assess their cytotoxicity. According to the research of Varshney et al. [65], a 4% SPI did not exert adverse effects on cells but instead promoted cell proliferation. This indicated that SPI, used in the preparation of SPIF, is safe, non-toxic, and serves as a healthy protein source. The cell viability status at various dilution concentrations of the samples was illustrated in Figure 17. Compared to the control group, SPIF concentrations ranging from 1.875 to 3.75 mg/mL had no significant effect on cell viability, with all values remaining above 95%. This indicated that the SPIF treated at prolonged low pH did not generate harmful substances, further confirming the safety. For the SPIF-TA and SPIF-GA hydrogels, cells viability exhibited normal survival across all concentration ranges, and even showed a slight enhancement. The results indicated that within a specific range, SPIF and SPIF–polyphenol complexes were safe and non-toxic within a specific range and do not adversely affect cell viability. Nevertheless, extensive experimentation was required to further validate the safety of SPIF and the SPIF–polyphenol complexes.

## 4. Conclusions

In this study, we investigated the effects of polyphenols (TA and GA) and their concentrations on the structure, gel characteristics, and biological activity of SPIF. The experimental results demonstrated that polyphenols remodel the structure of SPIF via hydrogen bonding and hydrophobic interactions, leading to the formation of polyphenol–amyloid hybrid nanofilaments. This enhanced both the gel properties and biological activity. Polyphenols deposited on the surface of SPIF result in thicker fibrils, denser structures, increased β-sheet content, and decreased random coil content. Initially, the free sulfhydryl group content slightly decreased, followed by a significant increase. Hydrogels exhibited a denser microstructure and enhanced gel strength. Notably, F-TA_20_ and F-GA_2_ displayed superior rheological properties, with storage moduli of 334.91 Pa and 317.79 Pa, respectively. The cohesion of these gels was significantly improved, reaching 49.18 Pa·s and 41.61 Pa·s, respectively, demonstrating the highest resistance to external environmental factors. Both SPIF and SPIF–polyphenol hydrogels exhibited effective antibacterial properties and safety. However, excessive polyphenols could adversely affect the structure and gel properties. Compared to GA, TA contained a higher number of hydroxyl groups and benzene rings, resulting in a stronger affinity for SPIF and a more pronounced effect on the structure and gel properties. These findings are valuable for selecting appropriate polyphenols, enhancing the understanding of their interaction mechanisms, and promoting their application in functional foods and pharmaceuticals.

## Figures and Tables

**Table 1:** Binding site (N), binding constant (KD), enthalpy change (ΔH), entropy change (ΔS), and Gibbs free energy (ΔG) for the interaction between SPI–polyphenol complexes (F-TA, F-GA). SPIF: soy protein fibril. TA: tannic acid. GA: gallic acid.

Samples	N (mol g^−1^, Protein Basis)	KD (M × 10^−4^)	ΔH (kcal mol^−1^)	−TΔS (kcal mol^−1^)	ΔG (kcal mol^−1^)
F-TA	0.0052 ± 0.0008 ^a^	2.39 ± 0.52 ^b^	−8.89 ± 1.22 ^a^	3.17 ± 0.42 ^b^	−5.72 ± 0.62 ^a^
F-GA	0.0024 ± 0.00014 ^b^	27.80 ± 1.37 ^a^	−2.36 ± 0.34 ^a^	5.85 ± 0.32 ^a^	−3.49 ± 0.39 ^b^

Means with the different superscript letters within the same column for each parameter are significantly different (*p* < 0.05).

**Table 2:** The relative composition of the water populations in SPIF, SPIF-AG, and SPIF-TA protein hydrogels. SPIF: soy protein fibril. TA: tannic acid. GA: gallic acid.

Sample	T_21_ (s)	P_21_ (%)	T_22_ (s)	P_22_ (%)	T_23_ (s)	P_23_ (%)
SPIF	1.589 ± 0.012 ^d^	0.499 ± 0.047 ^g^	310.787 ± 0.994 ^a^	99.501 ± 0.008 ^a^		
F-TA_50_	1.383 ± 0.008 ^e^	0.642 ± 0.038 ^f^	270.496 ± 1.421 ^c^	99.358 ± 0.006 ^b^		
F-TA_30_	1.482 ± 0.008 ^e^	0.696 ± 0.056 ^de^	270.496 ± 1.414 ^c^	99.304 ± 0.009 ^d^		
F-TA_20_	1.482 ± 0.014 ^e^	0.824 ± 0.033 ^b^	252.354 ± 1.226 ^d^	98.248 ± 0.003 ^i^	4994.505 ± 2.149 ^a^	0.928 ± 0.012 ^c^
F-TA_10_	1.047 ± 0.011 ^h^	0.679 ± 0.045 ^e^	219.639 ± 1.044 ^e^	97.369 ± 0.015 ^j^	2866.068 ± 3.147 ^c^	1.953 ± 0.014 ^a^
F-GA_50_	1.290 ± 0.009 ^f^	0.640 ± 0.019 ^f^	289.942 ± 1.195 ^b^	99.360 ± 0.002 ^b^		
F-GA_30_	1.203 ± 0.014 ^g^	0.652 ± 0.034 ^f^	289.942 ± 1.411 ^b^	99.348 ± 0.006 ^c^		
F-GA_20_	1.482 ± 0.021 ^e^	0.704 ± 0.022 ^d^	289.942 ± 1.254 ^b^	99.296 ± 0.002 ^e^		
F-GA_10_	1.383 ± 0.017 ^e^	0.763 ± 0.018 ^c^	289.942 ± 1.491 ^b^	99.237 ± 0.004 ^f^		
F-GA_5_	1.825 ± 0.015 ^c^	0.831 ± 0.047 ^b^	289.942 ± 1.672 ^b^	99.169 ± 0.002 ^g^		
F-GA_2_	1.956 ± 0.026 ^b^	0.835 ± 0.020 ^ab^	270.496 ± 0.858 ^c^	99.165 ± 0.003 ^h^		
F-GA_1_	2.409 ± 0.030 ^a^	0.853 ± 0.039 ^a^	252.354 ± 1.259 ^d^	97.397 ± 0.024 ^j^	3529.707 ± 5.174 ^b^	1.750 ± 0.024 ^b^

Means with the different superscript letters within the same column for each parameter are significantly different (*p* < 0.05).

**Table 3:** The rheological data of SPIF, SPIF-AG, and SPIF-TA protein hydrogels. SPIF: soy protein fibril. TA: tannic acid. GA: gallic acid.

Sample	K (Pa⋅s)	n	R^2^	G (Pa)	γmax (%)	Je (1/Pa) × 10^−2^	η (104 Pa s)
SPIF	16.26 ± 0.34 ^j^	0.3206 ± 0.0011 ^i^	0.9949 ± 0.0011 ^b^	68.61 ± 1.24 ^jk^	20.070 ± 0.221 ^a^	1.4580 ± 0.0044 ^a^	1.760 ± 0.97 ^hi^
F-TA_50_	32.40 ± 0.51 ^d^	0.3359 ± 0.0009 ^e^	0.9942 ± 0.0013 ^bc^	109.00 ± 2.64 ^h^	12.380 ± 0.384 ^d^	0.9171 ± 0.0071 ^d^	3.111 ± 0.221 ^f^
F-TA_30_	39.82 ± 0.39 ^c^	0.3561 ± 0.0023 ^c^	0.9932 ± 0.0017 ^c^	185.90 ± 1.79 ^e^	7.123 ± 0.224 ^f^	0.5380 ± 0.0096 ^g^	5.735 ± 0.239 ^d^
F-TA_20_	49.18 ± 0.58 ^a^	0.3748 ± 0.0031 ^a^	0.9927 ± 0.0010 ^d^	322.50 ± 5.27 ^a^	4.587 ± 0.107 ^h^	0.3101 ± 0.0071 ^j^	8.725 ± 0.307 ^a^
F-TA_10_	29.85 ± 0.69 ^e^	0.2870 ± 0.0017 ^j^	0.9985 ± 0.0016 ^a^	180.70 ± 2.58 ^f^	8.079 ± 0.254 ^e^	0.5533 ± 0.0081 ^f^	5.798 ± 0.357 ^d^
F-GA_50_	17.81 ± 0.34 ^i^	0.3219 ± 0.0024 ^hi^	0.9955 ± 0.0011 ^b^	69.61 ± 1.51 ^jk^	20.030 ± 0.254 ^ab^	1.4370 ± 0.0093 ^ab^	1.765 ± 0.083 ^hi^
F-GA_30_	21.00 ± 0.48 ^h^	0.3241 ± 0.0009 ^h^	0.9953 ± 0.0008 ^b^	71.48 ± 2.57 ^j^	19.670 ± 0.196 ^b^	1.3990 ± 0.0085 ^b^	1.819 ± 0.175 ^h^
F-GA_20_	22.18 ± 0.63 ^g^	0.3290 ± 0.0021 ^g^	0.9949 ± 0.0010 ^b^	104.80 ± 1.74 ^i^	13.350 ± 0.337 ^c^	0.9541 ± 0.0048 ^c^	2.621 ± 0.096 ^g^
F-GA_10_	27.54 ± 0.28 ^f^	0.3329 ± 0.0007 ^f^	0.9943 ± 0.0009 ^bc^	171.36 ± 3.28 ^g^	8.260 ± 0.315 ^e^	0.6014 ± 0.0054 ^e^	5.049 ± 0.353 ^e^
F-GA_5_	32.47 ± 0.85 ^d^	0.3494 ± 0.0015 ^d^	0.9936 ± 0.0010 ^c^	190.10 ±2.34 ^d^	6.794 ± 0.301 ^f^	0.5259 ± 0.0087 ^g^	6.507 ± 0.312 ^c^
F-GA_2_	41.61 ± 0.44 ^b^	0.3635 ± 0.0019 ^b^	0.9918 ± 0.0018 ^de^	264.70 ± 4.37 ^b^	4.952 ± 0.140 ^g^	0.3594 ± 0.0097 ^i^	7.364 ± 0.266 ^b^
F-GA_1_	40.80 ± 0.94 ^bc^	0.3618 ± 0.0020 ^b^	0.9903 ± 0.0007 ^e^	220.90 ± 5.31 ^c^	6.466 ± 0.297 ^fg^	0.4528 ± 0.0053 ^h^	5.513 ± 0.289 ^de^

Means with the different superscript letters within the same column for each parameter are significantly different (*p* < 0.05).

## Data Availability

The original contributions presented in this study are included in the article. Further inquiries can be directed to the corresponding authors.
